# Impact of single versus multiple infection on serum protein fractions in cats

**DOI:** 10.1007/s11259-025-10724-w

**Published:** 2025-04-04

**Authors:** Diana Marteles, María Eugenia Lebrero, Antonio Fernández, Aurora Ortín, Ana González, Carmen Morell, María Jesús Villanueva, Ingo Schäfer, Pablo Quílez, Maite Verde, Alex Gómez, Sergio Villanueva-Saz

**Affiliations:** 1 Clinical Immunology Laboratory, University of Zaragoza, Zaragoza, Spain; 2 Department of Animal Pathology, University of Zaragoza, Zaragoza, Spain; 3 Agro-food Institute of Aragón-IA2 (Universidad de Zaragoza-CITA), University of Zaragoza, Zaragoza, Spain; 4 HV, University of Zaragoza, Zaragoza, Spain; 5 LABOKLIN Gmbh and Co. KG, Steubenstraße 4, 97688 Bad Kissingen, Germany

**Keywords:** Cat, Serum protein electrophoresis, Coinfection, Globulin

## Abstract

Serum protein electrophoresis (SPE) is a widely used diagnostic tool for identifying acute and chronic inflammation, as well as immunodeficiencies. However, the impact of co-infections on SPE patterns in cats remains poorly understood. This study explored the utility of SPE in differentiating immune responses between cats infected with a single pathogen and those with multiple co-infections. A total of 79 serum samples from stray European Shorthair cats in Zaragoza, Spain, were analyzed. Fifty cats had a single infection, while 29 were co-infected with 2–4 pathogens. Agarose gel electrophoresis was used to assess protein profiles and statistical analyses were performed to identify significant differences between groups. The results showed no major differences in protein profiles between single and co-infected cats, with polyclonal hypergammaglobulinemia being most common in single-pathogen infections. Therefore, these findings indicate that SPE may have limitations in distinguishing between single and multiple infections in cats, contrasting with some previous studies.

**Supplementary Information:**

The online version contains supplementary material available at 10.1007/s11259-025-10724-w.

Serum protein electrophoresis (SPE) is a widely utilized diagnostic tool in both human and veterinary medicine. In human medicine is principally used in the diagnosis of monoclonal gammopathies, such as multiple myeloma (O’Connell et al. 2005). While a definitive diagnosis cannot be made solely based on SPE, it is useful in identifying and distinguishing between acute and chronic inflammation in veterinary medicine, as well as immunodeficiencies (Jania and Andraszek 2016; Moore and Avery 2019). Classic diagnostic categories used in veterinary medicine include electrophoretically physiological responses, acute-phase protein responses, polyclonal gammopathies, restricted polyclonal/oligoclonal gammopathies, and monoclonal gammopathies/paraproteinemias (Moore and Avery 2019).

The protein mixture is typically divided into six main fractions, including albumin, α1, α2, β1, β2 and γ globulins (Jania and Andraszek 2016). Mild increases in α1 and α2 globulins, and occasionally in β globulins, all these classified as acute-phase reactants, are characteristic of acute infections, inflammation, nephrotic syndrome, and malignancies (Taylor et al. 2010). A slight decrease in albumin is also common, as albumin is a negative acute-phase protein (Taylor et al. 2010; Jania and Andraszek 2016). In contrast, chronic infections and inflammations often result in elevated levels of γ immunoglobulins, composed mainly of immunoglobulins, particularly IgG and complement system, but also acute-phase proteins may increase (Taylor et al. 2010). An increase in γ globulins is indicative of sustained immune activation and prolonged antibody production (Jania and Andraszek 2016). Chronic conditions are also associated with a more pronounced reduction in albumin due to the ongoing inflammatory response and potential protein loss over time (Taylor et al. 2010; Moore and Avery 2019). For instance, cats infected with Feline Infectious Peritonitis (FIP) exhibit variable electrophoretic profiles (Sparkes et al. 1991), while those infected with Feline Leukemia Virus (FeLV) and Feline Immunodeficiency Virus (FIV) frequently present with polyclonal hypergammaglobulinemia (Miró et al. 2007). In other pathologies, including vector-borne infections such as *Anaplasma* spp. (Tarello 2005) or non-infectious conditions such as myeloma-related disorders (Mellor et al. 2006, 2008), it is possible to detect the presence of monoclonal gammopathies.

Coinfections tend to cause a more pronounced increase in 2 globulins and globulins, suggesting a more intense and sustained inflammatory and immunological response (Monteiro et al. 2010; De Tommasi et al. 2013; Baxarias et al. 2018; Asawakarn et al. 2021; Jornet-Rius et al. 2024). Recognizing these variations through SPE enhances diagnostic precision and informs more effective treatment strategies for infectious diseases in veterinary practice. Therefore, we hypothesize that animals co-infected with multiple systemic pathogens may exhibit different SPE values compared to those infected with a single pathogen.

The aim of this study was to evaluate serum protein concentration detected by SPE in cats infected with a single pathogen and those co-infected with multiple pathogens.

Seventy-nine serum samples from stray European shorthair cats were obtained in Zaragoza (Spain) from a previous prevalence study on feline vector-borne-pathogens (Villanueva-Saz et al. 2023). Cats were clinically evaluated, and polymerase chain reaction (PCR) testing of blood was performed specifically for detection of *Anaplasma spp.*, *Bartonella henselae*, *Ehrlichia canis*, *Rickettsia spp.*, *Mycoplasma spp.*, *Hepatozoon spp.*, *Leishmania infantum*, piroplasms, microfilariae, Feline Leukemia Virus (FeLV) and Feline Immunodeficiency Virus (FIV), as described in a previous study (Villanueva-Saz et al. 2023). Sera from cats presenting similar clinical signs with one detected infection (*n* = 50) and coinfections with 2–4 pathogens (*n* = 29) were selected for this study (Supplementary Table **S1**).

Serum protein electrophoresis was performed by agarose gel electrophoresis (AGE) (Hydragel Kit 1–2, Sebia, Issy-les-Moulineaux, France). Serum was electrophoresed for 21 min at 92 V and stained with diluted amidoschwarz dye at pH 2 (4 g/L amidoschwarz dye and 6.7% ethylene glycol). The AGE procedure was conducted according to the manufacturer’s instructions and commercial human serum was used as a control (normal control serum; Sebia, Evry, France). The electrophoretic curve for each sample was displayed and read with a GELSCAN TM densitometry system (Sebia, Issy-les-Moulineaux, France). The electrophoretic curve for each sample was assessed using Phoresis software. The software provided the number and location of each fraction´s at the same point in fraction. A manual adjustment of each fraction demarcation was assured for all samples by two different blinded independent examiners (Supplementary Figures S1, S2 and S3). Protein fractions were determined as a percentage of optical absorbance, and the absolute concentration g/dL was automatically calculated from the total serum protein concentration using a spectrophotometer. Albumin-to-globulin (A:G) ratios were also calculated. Reference intervals (RI) were previously described in Villanueva-Saz et al. 2024. Finally, total protein concentration was measured by the automatic analyzer Catalyst One Chemistry Analyzer (Idexx, USA). This is an in-clinic chemistry analyzer that operates using dry slide technology. Unlike wet chemistry analyzers, dry chemistry methods typically require less maintenance, making them well-suited for point-of-care testing in general veterinary practice. Additionally, this in-clinic dry chemistry analyzer features an internal centrifuge, enabling direct loading of whole blood, which further minimizes sample processing time (Boes et al. 2018).

Data were analyzed using IBM SPSS 28.0 for Windows^®^. Serum protein electrophoresis values were described using mean and standard deviation (SD). After testing normal distribution of the data by Shapiro–Wilk test, for normally distributed variables Levene’s test was applied. For variables with equal variances, one-way analysis of variance (ANOVA) was applied. After ANOVA analysis, Bonferroni correction was applied in multiple pairwise comparisons. For normal variables with unequal variances, Welch’s t-test was used and multiple pairwise comparisons were performed using Games Howell Post-hoc test. For non-normal distributed variables, Kruskal–Wallis test was conducted and multiple pairwise comparisons were applied by Dunn post-hoc test. To compare the serum protein electrophoresis values within the same group, paired Sample T-Test (normal variables) or Wilcoxon test (non-normal variables) were used. The α-error was set at 0.05.

Cats infected with one pathogen (3.48; 95% CI 3.12–3.83%) presented lower percentage of α1 globulin than the group infected with two pathogens (4.2; 95% CI 3.84–4.56) (*p* = 0.022) and lower quantity of γ globulin (1.23; 95 CI 0.97–1.48 g/dL) than the group of cats infected with 4 pathogens (2.37; CI 95% 0.94–5.67 g/dL) (*p* = 0.044). Cats infected with 2 pathogens had lower percentage (17.02; 95% CI 14.36–19.68%) and quantity (1.21; CI 95% 0.87–1.54 g/dL) of γ globulin than cats infected with 4 pathogens (27.5; CI 95% 1.78–53.2%, 2.37; CI 95% 0.94–5.67 g/dL) (*p* = 0.013, *p* = 0.028). However, no statistically significant differences were observed in any value of the SPE between cats infected with one pathogen and cats coinfected with more than one pathogen (Supplementary Table S2).

Additionally, in cats infected with a single pathogen, α2 globulin was the highest percentage (17.38; 95% CI 15.6–19.16) in the SPE (Fig. 1), while γ globulin was the largest quantity (1.23; 95% CI 0–97-1.48 g/dL) (Fig. 2). The percentage of α2 globulin (*p* < 0.001) and the quantity of γ globulin (*p* = 0.001) were significantly higher than α1 globulin (3.48; 95% CI 3.12–3.83, 0.23; 95% CI 0.20–0.26). In cats coinfected with multiple pathogens, γ globulin was the most abundant globulin (19; 95% CI 16.45–21.54%, 1.35; 95% CI 1.05–1.65) (Figs. 1 and 2). The percentage and quantity of γ globulin was significantly higher than α1 globulin (3.98; 95% CI 3.7–4.26%, 0.26; 95% CI 0.22–0.3 g/dL) (*p* = 0.004, *p* = 0.028), although this finding can be observed also in healthy cats. The albumin/globulin ratio was higher in cats with a single infection (0.97) compared to those with multiple infections (0.9) (Supplementary Table S2).

In this study, SPE was conducted on cats infected with a single pathogen and those coinfected with 2 to 4 pathogens to assess differences in their electrophoretic patterns. Despite the expectation of more complex immune responses in coinfected animals, the overall protein profiles were surprisingly similar across both groups. Protein fractions can vary depending on the pathogen involved (Jornet-Rius et al. 2024), which suggests that different immune pathways might counterbalance one another, resulting in globulin increases similar to those seen in single-pathogen infections. Furthermore, the immune response to each pathogen might be relatively minor, leading to a similar protein profile in both single and multiple infections (Milanović et al. 2020). If the coinfecting pathogens are of similar types (e.g., both parasitic), the immune response may not differ sufficiently to cause distinguishable changes in protein fractions (Cloete et al. 2024). However, in this study, cats infected with bacteria, viruses, and protozoa exhibited comparable electrophoretic patterns, indicating that SPE may not reliably distinguish between single and multiple infections in feline patients.

Both groups showed increases in total proteins and α2 globulin levels, with the single-pathogen group also exhibiting a slight increase in β1 globulin compared to the RI (Villanueva-Saz et al. 2022). In cats infected with a single pathogen, there was a significant rise in both α2 and γ globulins relative to other globulin fractions. This pattern aligns with a typical acute-phase response to infection (Taylor et al. 2010). α2 globulins are produced in response to inflammation and tissue damage, and their levels are expected to peak within 24–48 h and resolve within 4‐7 days after a single initial stimulus (Petersen et al. 2004; Moore and Avery 2019). Therefore, the results suggest that the cats studied were experiencing acute infections (Salt 1956), which is in accordance with positive PCR testing. Additionally, the observed increases in α2 and γ globulins suggest a balanced immune response, involving both the innate immune system (reflected by α2 proteins) and the adaptive immune system (indicated by γ globulins) (Porter 1960; Vandooren and Itoh 2021). Although γ globulins remained within the RI in both groups, cats coinfected with more than two pathogens showed the highest elevations in the γ globulin fraction. This rise in γ globulins, primarily consisting of immunoglobulins, indicates an active immune response to the pathogen (Porter 1960). Immunoglobulins are crucial for neutralizing pathogens, and their production correlates with the type and severity of infection. Based on literature, co-infections can stimulate a more robust adaptive immune response, requiring simultaneous responses to multiple antigens and leading to greater immunoglobulin production. This can shift protein fractions, with the γ globulin fraction becoming dominant (Taylor et al. 2010; De Tommasi et al. 2013; Jornet-Rius et al. 2024).

The results of this study indicate that acute infections in cats can exhibit a range of electrophoretic patterns, with polyclonal hypergammaglobulinemia being the most common in single-pathogen infections, while α2 globulin increase was more frequently observed in coinfected animals. This is in contrast with previous studies, where both infected and coinfected cats and dogs generally showed polyclonal increases in γ and/or β globulins (Taylor et al. 2010; Baxarias et al. 2018; Asawakarn et al. 2021; Jornet-Rius et al. 2024). In addition, the albumin/globulin ratio remained within reference values in both groups, differing from other studies (Baxarias et al. 2018; Asawakarn et al. 2021).

In conclusion, this study specifically focuses on SPE results in cats, examining a large retrospective sample of stray cats from a referral population, and describing differences between cats infected with a single pathogen versus those with multiple infections. In summary, our findings demonstrate that serum protein fractions do not differ between cats with single and multiple infections. This consistency in SPE patterns can help simplify diagnostic procedures, providing a valuable tool for evaluating inflammation and immune responses in cats, ultimately enhancing the accuracy and efficiency of disease diagnosis and management in veterinary practice. Further studies on hematological and biochemical parameters are required to enhance understanding of this topic.

**Fig. 1:**
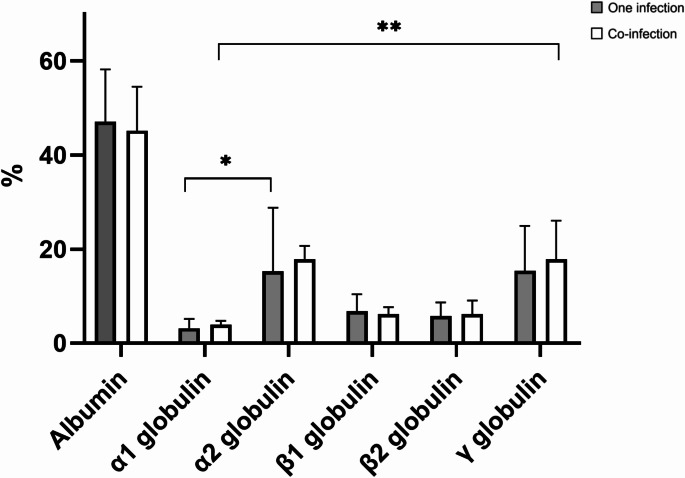
Comparison of the percentage of each protein fraction between cats with a single infection and cats with multiple co-infections

**Fig. 2:**
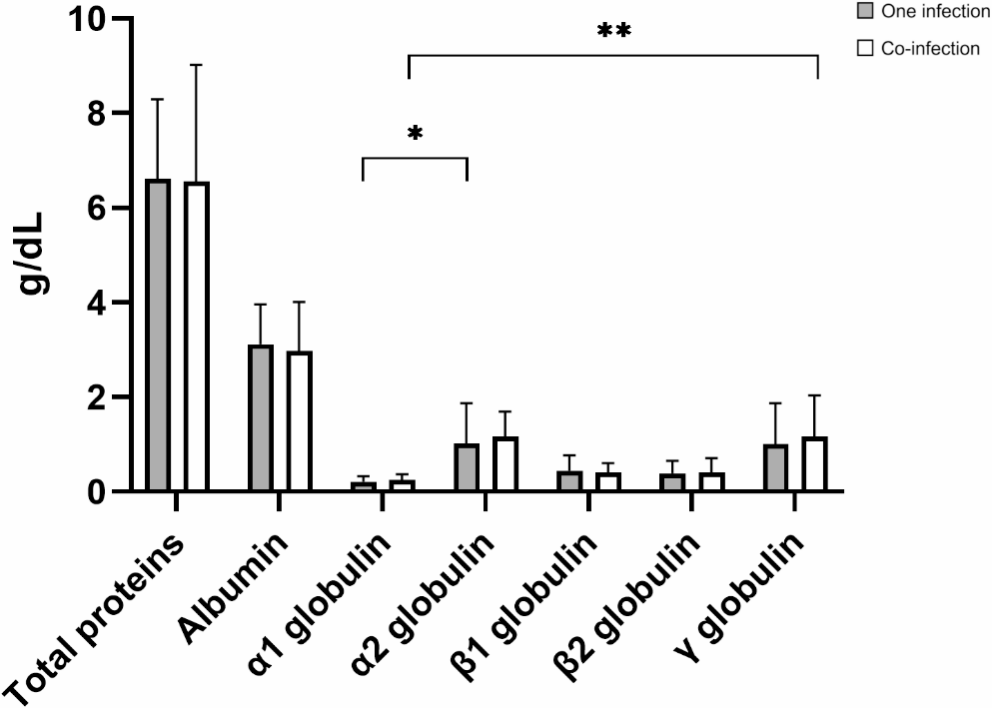
Comparison of the concentration of each protein fraction between cats with a single infection and cats with multiple co-infections

## Electronic supplementary material

Below is the link to the electronic supplementary material.

Supplementary Material 1

ESM 1 (PNG 616 KB)

High Resolution Image (TIF 143 KB)

ESM 2 (PNG 609 KB)

High Resolution Image (TIF 141 KB)

ESM 3 (PNG 433 KB)

High Resolution Image (TIF 136 KB)

## Data Availability

The datasets generated during and/or analyzed during the current study are available from the corresponding author on reasonable request.
