# Supplementary material for: Impact of single versus multiple infection on serum protein fractions in cats
Source: Vet Res Commun. 2025 Apr 4;49(3):158. doi: 10.1007/s11259-025-10724-w (PMC11971170; doi:10.1007/s11259-025-10724-w)
Supplement: Supplementary file 1 — Supplementary Material 1 [file 11259_2025_10724_MOESM1_ESM.docx]

**Supplementary material**

**Brief report**: Impact of single versus multiple infection on serum protein fractions in cats

Diana Marteles^1,2,3^, María Eugenia Lebrero^1,4^, Antonio Fernández^1,2,3^, Aurora Ortín^2,3^, Ana González^1,4^, Carmen Morell^4^, María Jesús Villanueva^4^, Ingo Schäfer^5^, Pablo Quílez^1^, Maite Verde^1,2,3^, Álex Gómez^1^*, Sergio Villanueva-Saz^1,2,3*^

^1^ Clinical Immunology Laboratory, Veterinary Faculty, University of Zaragoza, 50013 Zaragoza, Spain

^2^ Department of Animal Pathology, Veterinary Faculty, University of Zaragoza, Zaragoza, Spain

^3^ Instituto Agroalimentario de Aragón-IA2 (Universidad de Zaragoza-CITA), Zaragoza, Spain

^4^ H.V.U.Z., Veterinary Faculty, University of Zaragoza, 50013 Zaragoza, Spain

^5^ LABOKLIN GmbH and Co. KG, Steubenstraße 4, 97688 Bad Kissingen, Germany.

***Corresponding author:**

Sergio Villanueva-Saz and Álex Gómez (0000-0002-9723-9004)

Emails: [svs@unizar.es](mailto:svs@unizar.es) and [a.gomez@unizar.es](mailto:a.gomez@unizar.es)

**Supplementary Table S1**. PCR detected pathogens in the blood of stray cats from Zaragoza, Spain.

| Nº infections | Pathogen | Affected cats |
| --- | --- | --- |
| 1 | MHF | 2.5% (n=2) |
|  | MHM | 11.4% (n=9) |
|  | Hep | 36.7% (n=29) |
|  | Leish | 1.3% (n=1) |
|  | BH | 7.6% (n=6) |
|  | FeLV | 3.8% (n=3) |
| 2 | MHM + Hep | 1.3% (n=1) |
|  | MHM + MHF | 5% (n=4) |
|  | MHM + BH | 1.3% (n=1) |
|  | MHM + FeLV | 2.5% (n=2) |
|  | FeLV + FIV | 1.3% (n=1) |
|  | Hep + BH | 6.3 (n=5) |
|  | MHF + Hep | 3.8% (n=3) |
|  | BH + Leish | 1.3% (n=1) |
|  | Hep + MT | 1.3% (n=1) |
| 3 | MHF + MHM + Hep | 5% (n=4) |
|  | BH + FeLV + FIV | 1.3% (n=1) |
|  | MHM + MT + Leish | 2.5% (n=2) |
| 4 | MHF + MHM + MT + BH | 1.3% (n=1) |
|  | MHM + MT + BH + Leish | 1.3% (n=1) |
|  | MHM + Leish + FeLV + FIV | 1.3% (n=1) |

Abbreviations: MHF: *Mycoplasma haemofelis*; MHM: *Mycoplasma haemominutum*; MT: *Mycoplasma turicensis*; Hep: *Hepatozoon spp*.; Leish: *Leishmania infantum*; FeLV: *Feline Leukemia Virus*; BH: *Bartonella henselae*; FIV: *Feline Immunodeficiency Virus*.

**Supplementary Table S2**. Serum protein electrophoresis values from stray cats from Zaragoza, Spain infected with 1 or 2-4 vector-borne pathogens.

| Proteinogram values |  | One infection | Co-infection | RI | *p* |
| --- | --- | --- | --- | --- | --- |
| Total proteins (g/dL) | Mean | 6.79 | 6.88 | 5.7-5.9 | 0.833 |
|  | SD | 1.54 | 2.13 |  |  |
| Albumin (%) | Mean | 47.98 | 45.97 | 45-60 | 0.326 |
|  | SD | 8.82 | 8.57 |  |  |
| Albumin (g/dL) | Mean | 3.20 | 3.10 | 2.1-4.0 | 0.619 |
|  | SD | 0.76 | 0.89 |  |  |
| α1 globulin (%) | Mean | 3.48 | 3.99 | 4-14 | 0.505 |
|  | SD | 1.25 | 0.74 |  |  |
| α1 globulin (g/dL) | Mean | 0.23 | 0.25 | 0.1-1.1 | 0.177 |
|  | SD | 0.09 | 0.11 |  |  |
| α2 globulin (%) | Mean | 17.38 | 18.06 | 7-12 | 0.584 |
|  | SD | 6.26 | 2.73 |  |  |
| α2 globulin (g/dL) | Mean | 1.17 | 1.25 | 0.4-0.9 | 0.475 |
|  | SD | 0.48 | 0.46 |  |  |
| β1 globulin (%) | Mean | 7.59 | 6.36 | 7-15.5 | 0.146 |
|  | SD | 4.39 | 1.38 |  |  |
| β1 globulin (g/dL) | Mean | 0.53 | 0.45 | 0.15-0.45 | 0.264 |
|  | SD | 0.39 | 0.19 |  |  |
| β2 globulin (%) | Mean | 6.29 | 6.62 | 7-16 | 0.628 |
|  | SD | 3.14 | 2.45 |  |  |
| β2 globulin (g/dL) | Mean | 0.44 | 0.47 | 0.15-0.49 | 0.654 |
|  | SD | 0.27 | 0.24 |  |  |
| γ globulin (%) | Mean | 17.27 | 19.00 | 10-28 | 0.3698 |
|  | SD | 9.00 | 6.69 |  |  |
| γ globulin (g/dL) | Mean | 1.23 | 1.36 | 1.2-2.2 | 0.543 |
|  | SD | 0.91 | 0.79 |  |  |
| Albumin/globulin ratio | Mean | 0.97 | 0.90 | 0.45-1.3 | 0.326 |
|  | SD | 0.31 | 0.33 |  |  |

Abbreviation: RI: reference intervals.

**Supplementary Figure S1.** Example of representative tracing to demonstrate where fraction demarcations were made**.**

**Supplementary Figure S2.** Example of representative tracing to demonstrate where fraction demarcations were made**.**

**Supplementary Figure S3.** Example of representative tracing to demonstrate where fraction demarcations were made**.**
