# Supplementary figures and images for: Impact of single versus multiple infection on serum protein fractions in cats
Source: Vet Res Commun. 2025 Apr 4;49(3):158. doi: 10.1007/s11259-025-10724-w (PMC11971170; doi:10.1007/s11259-025-10724-w)

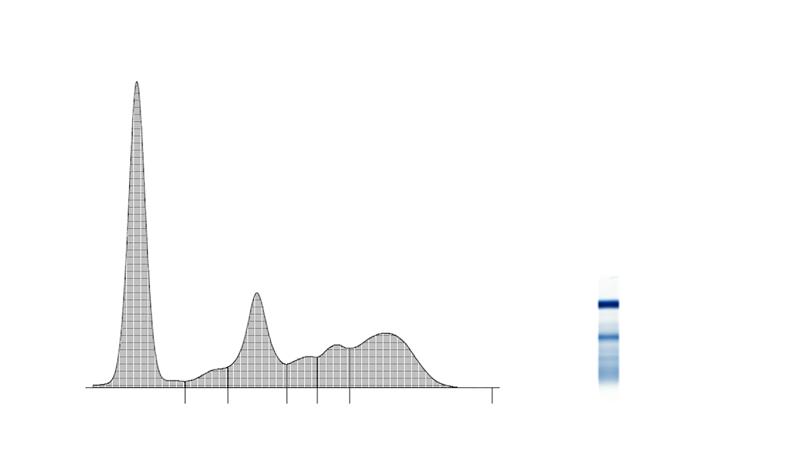

Supplement: Supplementary file 2 — (PNG 616 KB) [file 11259_2025_10724_Fig3_ESM.png]

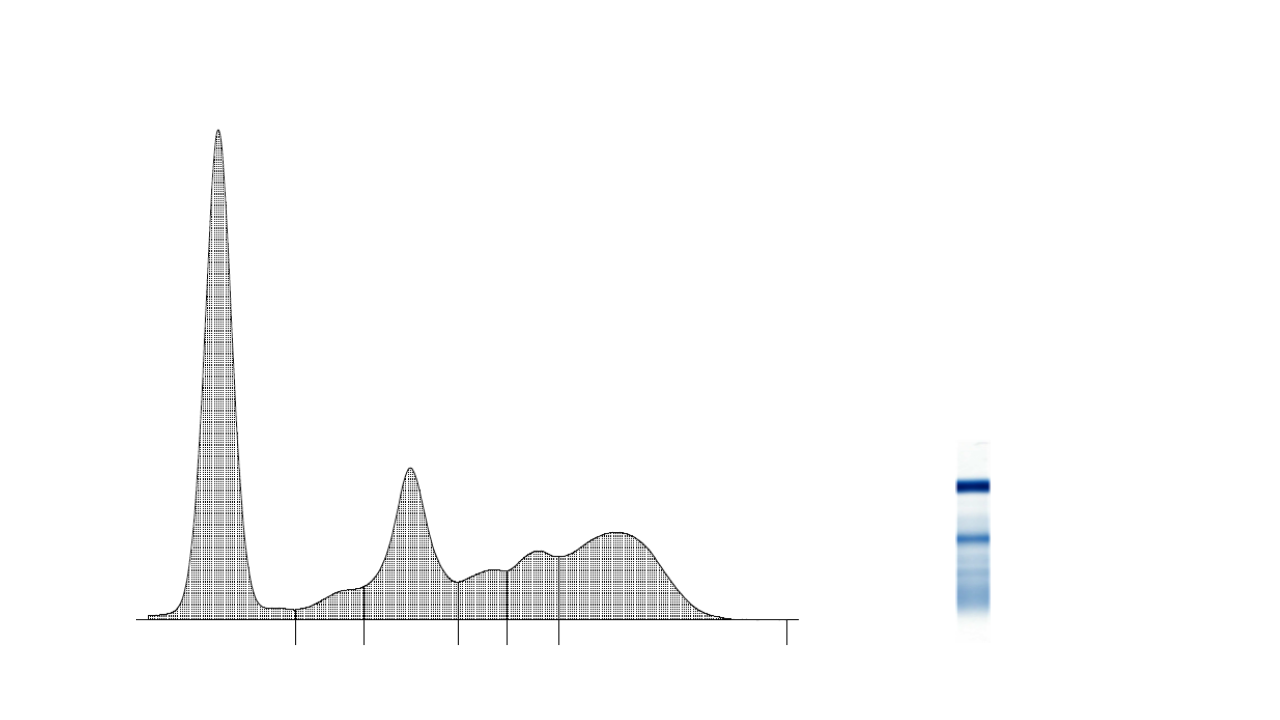

Supplement: Supplementary file 3 — High Resolution Image (TIF 143 KB) [file 11259_2025_10724_MOESM2_ESM.tif]

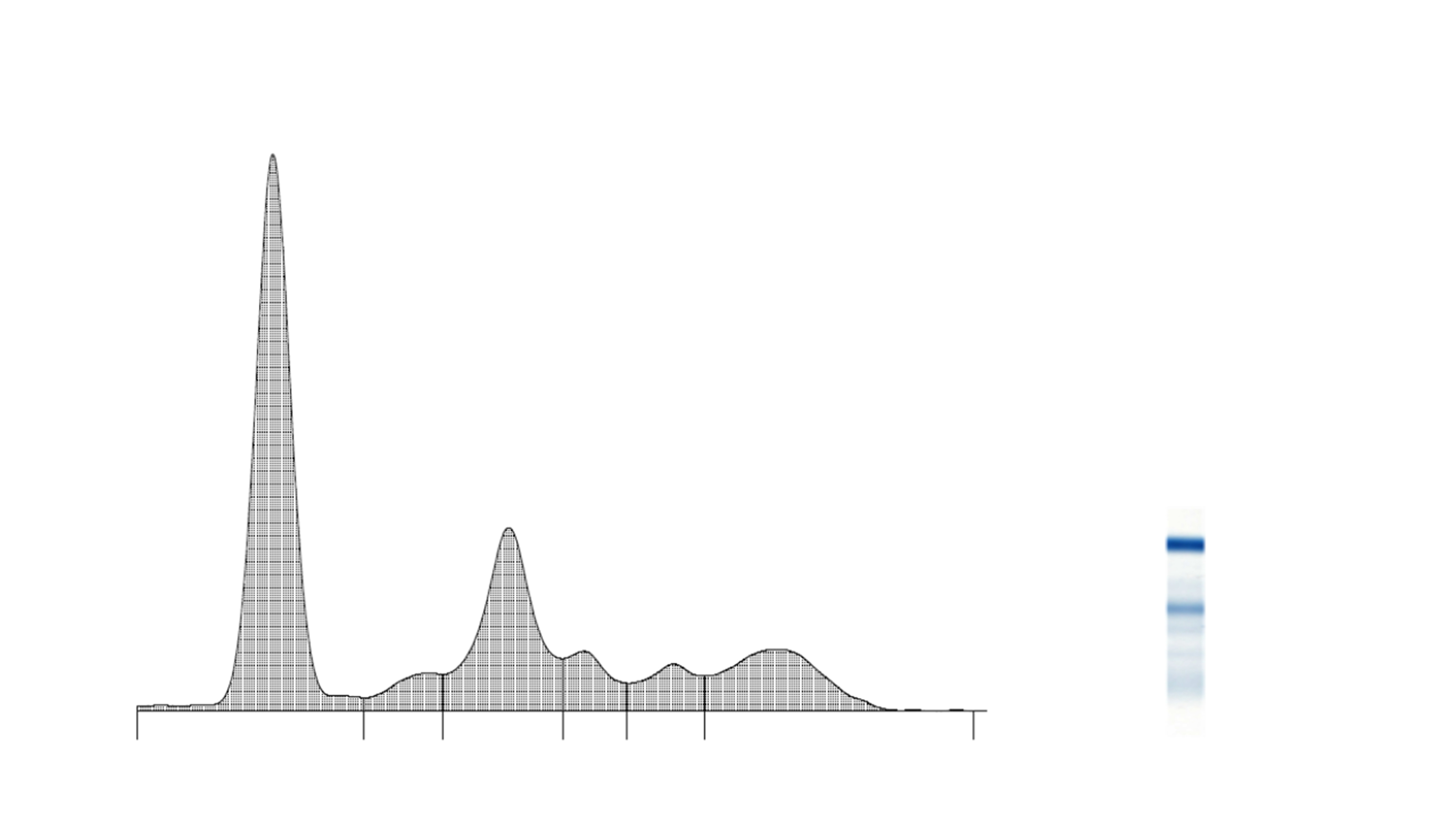

Supplement: Supplementary file 4 — (PNG 609 KB) [file 11259_2025_10724_Fig4_ESM.png]

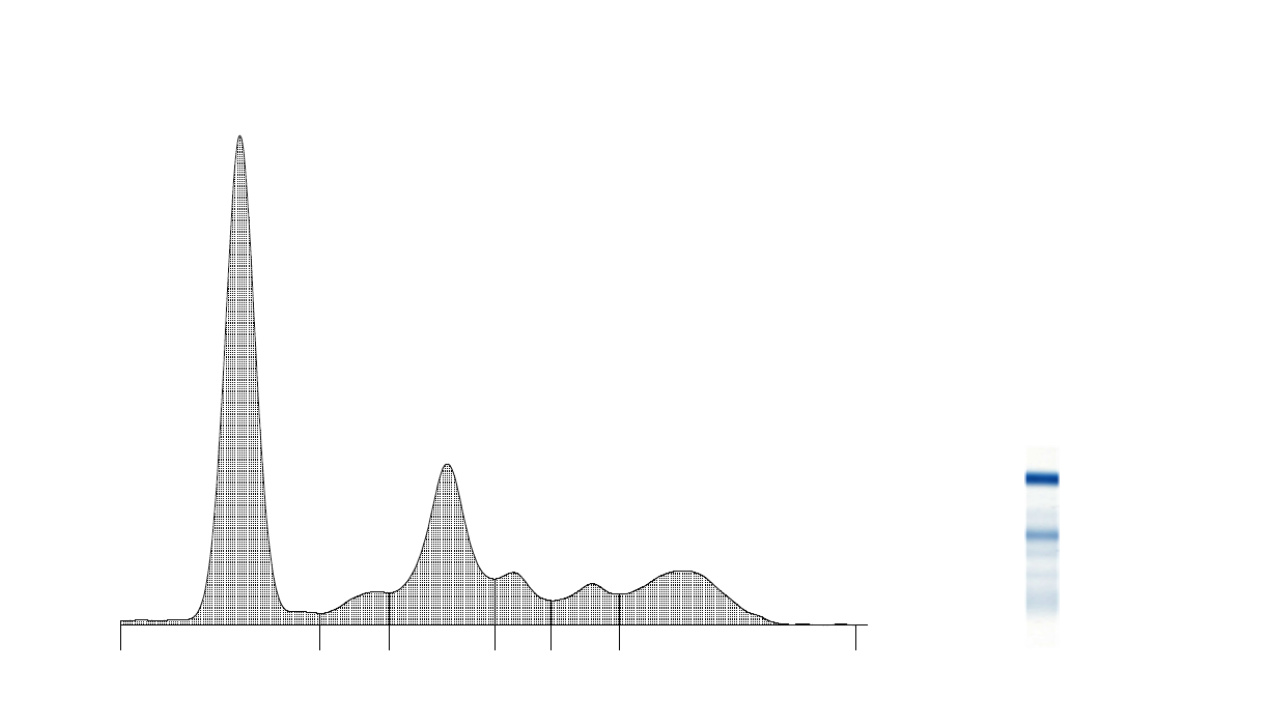

Supplement: Supplementary file 5 — High Resolution Image (TIF 141 KB) [file 11259_2025_10724_MOESM3_ESM.tif]

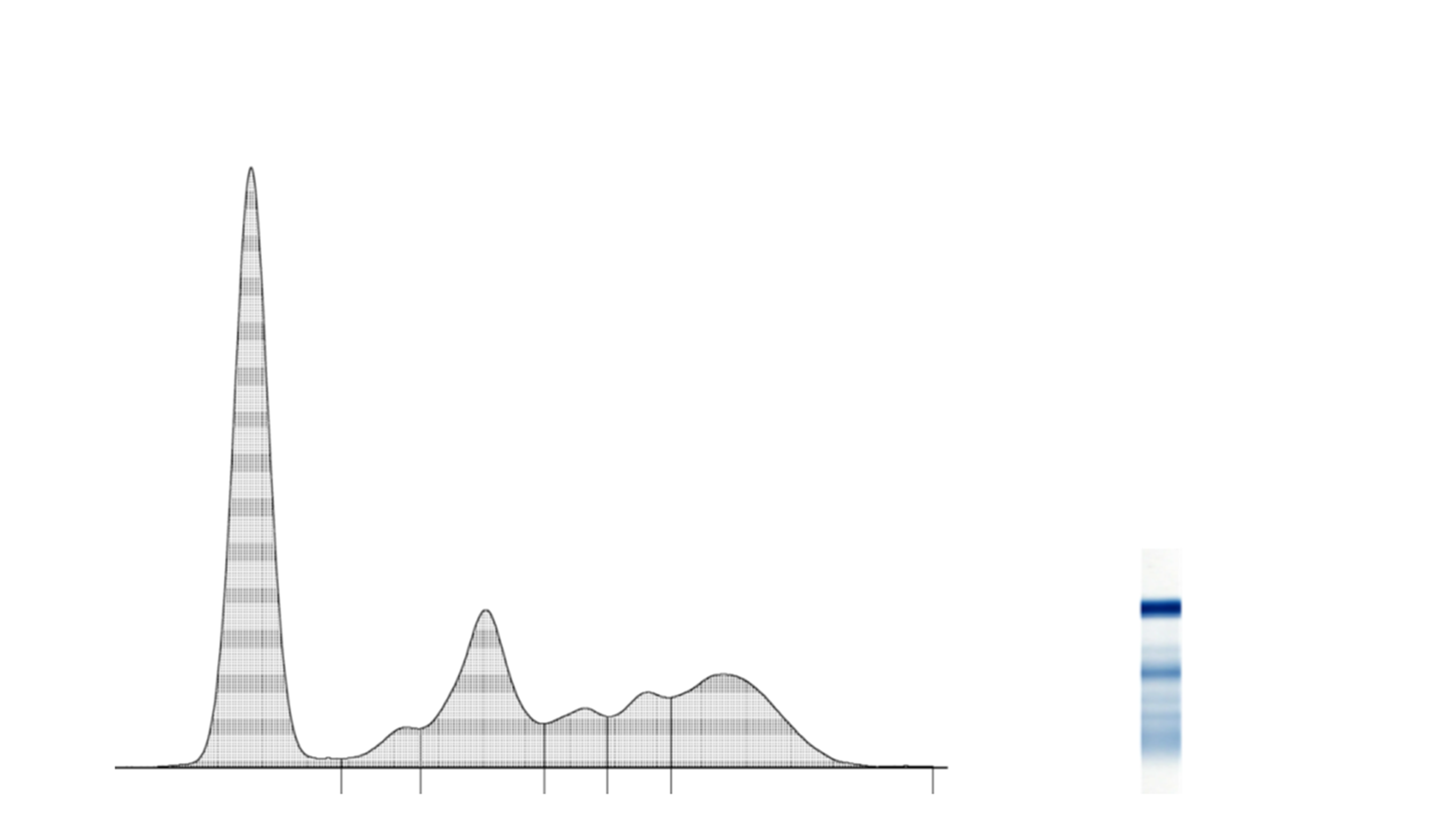

Supplement: Supplementary file 6 — (PNG 433 KB) [file 11259_2025_10724_Fig5_ESM.png]

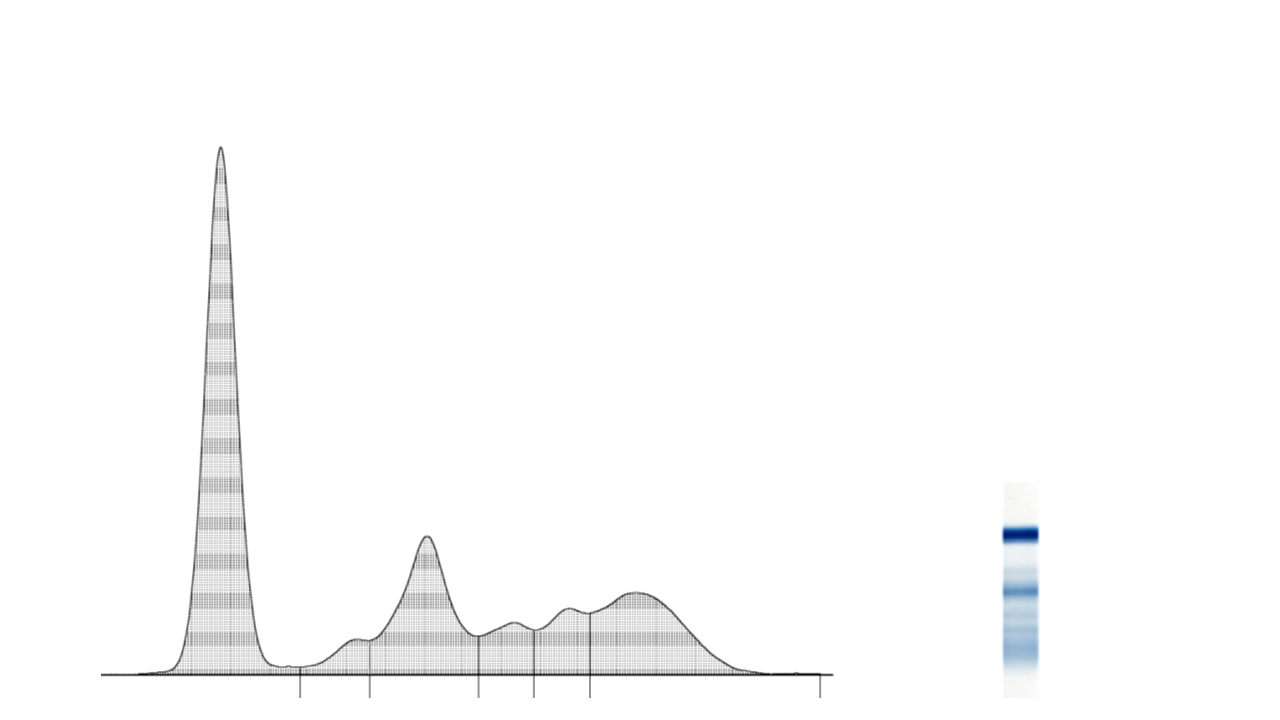

Supplement: Supplementary file 7 — High Resolution Image (TIF 136 KB) [file 11259_2025_10724_MOESM4_ESM.tif]
